# The Presence/Absence of an Awake-State Dominant EEG Rhythm in Delirious Patients Is Related to Different Symptoms of Delirium Evaluated by the Intensive Care Delirium Screening Checklist (ICDSC)

**DOI:** 10.3390/s24248097

**Published:** 2024-12-19

**Authors:** Toshikazu Shinba, Yusuke Fujita, Yusuke Ogawa, Yujiro Shinba, Shuntaro Shinba

**Affiliations:** 1 Department of Psychiatry, Shizuoka Saiseikai General Hospital, Shizuoka 422-8527, Japan; 2 Research Division, Saiseikai Research Institute of Health Care and Welfare, Tokyo 108-0073, Japan; 3 Ward South 8, Shizuoka Saiseikai General Hospital, Shizuoka 422-8527, Japan; 4 Intensive Care Unit, Ward East 6, Shizuoka Saiseikai General Hospital, Shizuoka 422-8527, Japan; 5 Autonomic Nervous System Consulting, Shizuoka 420-0839, Japan; 6 Department of General Medicine, Shizuoka Saiseikai General Hospital, Shizuoka 422-8527, Japan

**Keywords:** delirium, electroencephalography, dominant rhythm, eyes opening and closing, Intensive Care Delirium Screening Checklist, hallucination or delusion, symptom fluctuation

## Abstract

(1) Background: Delirium is a serious condition in patients undergoing treatment for somatic diseases, leading to poor prognosis. However, the pathophysiology of delirium is not fully understood and should be clarified for its adequate treatment. This study analyzed the relationship between confusion symptoms in delirium and resting-state electroencephalogram (EEG) power spectrum (PS) profiles to investigate the heterogeneity. (2) Methods: The participants were 28 inpatients in a general hospital showing confusion symptoms with an Intensive Care Delirium Screening Checklist (ICDSC) score of 4 or above. EEG was measured at Pz in the daytime awake state for 100 s with the eyes open and 100 s with the eyes closed on the day of the ICDSC evaluation. PS analysis was conducted consecutively for each 10 s datum. (3) Results: Two resting EEG PS patterns were observed regarding the dominant rhythm: the presence or absence of a dominant rhythm, whereby the PS showed alpha or theta peaks in the former and no dominant rhythm in the latter. The patients showing a dominant EEG rhythm were frequently accompanied by hallucination or delusion (*p* = 0.039); conversely, those lacking a dominant rhythm tended to exhibit fluctuations in the delirium symptoms (*p* = 0.020). The other ICDSC scores did not differ between the participants with these two EEG patterns. (4) Discussion: The present study indicates that the presence and absence of a dominant EEG rhythm in delirious patients are related to different symptoms of delirium. Using EEG monitoring in the care of delirium will help characterize its heterogeneous pathophysiology, which requires multiple management strategies.

## 1. Introduction

Delirium is a serious problem in the treatment of somatic diseases, leading to poor prognosis [1]. Delirious symptoms restrict normal daily activity and exacerbate clinical conditions by reducing communication, decreasing nutrition, and necessitating sedation. Treatment procedures are also significantly hindered by delirious symptoms. It is important to adequately detect and ameliorate delirious symptoms to ensure recovery from the underlying diseases.

Delirium manifests a wide range of symptoms in somatic disease processes, including the disturbances of consciousness, attention, and cognition alongside their diurnal fluctuation [2]. Symptom checklists have often been utilized for detecting delirium in clinical practice, including the Confusion Assessment Method [3], the Richmond Agitation–Sedation Scale [4], and the Intensive Care Delirium Screening Checklist (ICDSC) [5]. The total scores of these clinical scales are used to diagnose delirium.

It is necessary to understand delirium’s pathophysiological heterogeneity in its diagnosis [6]. Various factors underlie the etiology of delirium, requiring different treatments. Previous research indicates that subclassification is important to treat and manage delirious states, including hyperactive and hypoactive delirium. Patients with hyperactive and hypoactive delirium show different clinical manifestations: the former exhibit psychomotor symptoms, while the latter have more serious somatic conditions and long hospitalization [7]. Hallucination and delusion are more frequently observed in hyperactive delirium than in hypoactive delirium [8]. Persistence of hypoactive delirium will lead to worsening cognitive function [9].

Despite these discrete clinical manifestations, no etiological differences between these two subtypes have been noted [7]. Furthermore, it is reported that hallucination and delusion are also present in hypoactive delirium, although their prevalence is lower than in hyperactive delirium. The presence of these symptoms is insufficient to distinguish between these two types of delirium [8]. Delineating delirium subtypes is important in assessing the intervention and evaluating the prognosis. Further research is warranted to clarify the heterogeneity and investigate the adequate therapies for the delirium subtypes [9].

This study aimed to clarify the heterogeneity of delirious states by incorporating electroencephalographic recordings as the objective measure to evaluate the pathophysiology of delirium, in addition to a checklist. Electroencephalograms (EEGs) have been thoroughly studied for characterizing the brain condition in delirious patients. Compared with a normal brain condition, delirium is frequently accompanied by diffuse slowing with increases in theta and delta power and reductions in alpha power [10,11,12,13,14,15]. These features were clarified quantitatively as the absolute power as well as the ratios of that for the two frequency bands. The shift of peak to a lower frequency range was also noted. In addition to slowing EEG rhythms, a diminution in responses to eye opening is also observed [12]. Furthermore, an absence of a dominant rhythm is reported in patients with delirium [12]. The presence of various EEG changes in delirium suggests that EEG monitoring helps diagnose delirium [16,17,18,19,20,21] and verify the risk of delirium [22,23]. EEG recordings with a small number of electrodes and automatic assessment were employed in addition to standard EEG monitoring. It has been suggested that EEG recording is informative in assessing delirium alongside regular neurocognitive evaluation and inflammatory examinations [24,25].

Based on these findings regarding the heterogeneity of delirium and various EEG changes, we analyzed the relationship between delirium symptoms and the patterns of EEG recordings to examine the heterogeneity. The EEGs were recorded on the head surfaces of delirious patients, and the relationship between the EEG patterns and delirium symptoms was analyzed using the symptom evaluation with ICDSC [5].

## 2. Materials and Methods

### 2.1. Participants

The participants included 28 inpatients (age: 75.4 ± 12.0 years; mean ± s.d.; 14 men and 14 women) attending Shizuoka Saiseikai General Hospital, showing delirious symptoms with an ICDSC score of 4 or above. They were under treatment for somatic disorders. The number of patients with cardiovascular, infectious, neoplastic, orthopedic, metabolic, neurological, or hepatorenal disorders were 2, 3, 4, 4, 3, 6, and 6, respectively. They were consecutively enrolled from August 2017 to August 2018 by consultation between the attending doctors and a psychiatrist for the care of confusion. Unconscious patients who could not respond to the instruction to open and close their eyes were excluded.

The ICDSC was scored on the day of consultation. The presence of altered levels of consciousness, inattention, disorientation, hallucination or delusion, psychomotor agitation or retardation, inappropriate mood or speech, sleep/wake cycle disturbance, and symptom fluctuation was scored as 1 when the symptom was present [5]. The sum of the eight scores was used as the total ICDSC score.

No psychiatric medication was used on the day and the previous day of the EEG measurements. Written informed consent was obtained from the patients or their families to participate in the present clinical research study. The protocol of this study was approved by the Institutional Review Board of Shizuoka Saiseikai General Hospital (no. 23-8-02).

### 2.2. EEG Measurements

The EEGs were measured at Pz with an international 10/20 system in the daytime awake state on the bed for 100 s with the eyes open and 100 s with the eyes closed on the day of the ICDSC evaluation. The measurement site at Pz was chosen because the posterior alpha wave was readily recorded, and the EEG electrode placement caused little distress to the patients lying on a hospital bed with various somatic disorders. The reference electrode was placed on the earlobe. A portable 16-bit amplifier was used for the EEG measurements (MWM-12, GMS, Tokyo, Japan). A bandpass filter of 0.1–30 Hz was employed. The EEG signals were stored at a sampling frequency of 200 Hz in a computer for the power spectrum analysis using the Mem-Calc software (GMS, Tokyo, Japan). The EEG power spectrum (PS) analysis was conducted consecutively for each 10 s datum using software attached to the amplifier (MWM-12, GMS, Tokyo, Japan), and the powers of EEG bands—delta (0.5–4 Hz), theta (4–8 Hz), alpha (8–13 Hz), and beta (13–20 Hz) bands—were calculated.

We confirmed the validity of EEG recording by verifying the following points. The electrode impedance was checked before each recording and was below 100 k Ohm to exclude the recordings with inappropriate noise; some parts of power spectral density in the range of 0.5–20 Hz were larger than 1 μV^2^/Hz, indicating that EEG exhibited fluctuations in that range, and no peak was present in the range of 20–30 Hz, reflecting electromyogram, suggesting the presence of movement artifacts. These technical points ensuring the validity of EEG recording were based on the conventional literature [26]. We also confirmed the validity of the system by monitoring electrocardiograms using the same device simultaneously with EEG. EEG recording by a similar device was also properly used in our previous publication [27].

### 2.3. Statistical Analysis

Two-way repeated-measures analysis of variance (ANOVA) with post hoc Sidak multiple comparisons was used to analyze the EEG amplitude power of the delta, theta, alpha, and beta bands in the eyes-open and -closed conditions of the participants with different EEG profiles. The total ICDSC scores and gender distributions were compared using the Mann–Whitney U test. Fisher’s exact test was employed for each ICDSC score. The differences in the distributions of the underlying diseases between the two groups of participants were checked using the chi-square test (Prism 8, Version 8.4.3, GraphPad Software, San Diego, CA, USA).

## 3. Results

### 3.1. EEG Patterns Regarding the Dominant Rhythms

**Figure 1:**
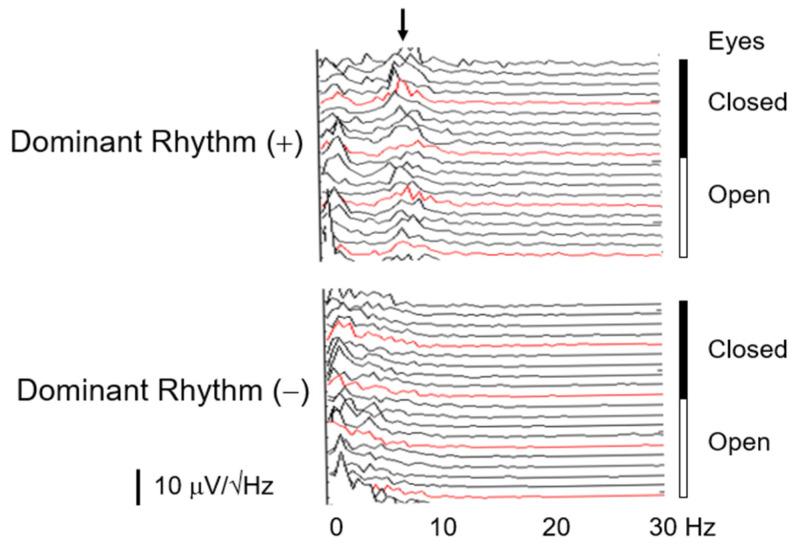
Different types of EEG power spectrum in sequential 10 s data at the parietal head position (Pz) in the eyes-open (Open) and -closed (Closed) conditions in two delirious patients. The data in the red line were inserted at the interval of 50 s. The arrow indicates the power spectrum peak at 8 Hz in a participant showing a dominant EEG rhythm (Dominant Rhythm (+)). No peak is present in the power spectrum of a participant without a dominant EEG rhythm (Dominant Rhythm (−)).

Two EEG patterns were observed regarding the presence (n=12) or absence (n=16) of dominant rhythms: dominant (+) and dominant (−), as shown in Figure 1. In this study, the presence of dominant rhythm was determined by the presence of a peak in the PS for the total measurement time with the differences in power from the preceding and following inflection points being more than 50% of the power at these points. Figure 1 presents two types of EEG power spectrum: the presence or absence of dominant rhythms, dominant (+) and dominant (−), in sequential 10 s data at the parietal head position in the eyes-open and -closed conditions in two delirious patients. The age and gender distributions were 79.3 ± 8.9 years (mean ± s.d.) and 4 men/8 women in the patients with the former pattern, and 72.5 ± 13.5 and 10 men/6 women in the patients with the latter. No age (Mann–Whitney U test: ±U = 70; *p* = 0.2357) nor gender (Fisher’s exact test; *p* = 0.2519) differences were noted.

Regarding the main underlying disease, the numbers of patients with cardiovascular, infectious, neoplastic, orthopedic, metabolic, neurological, or hepatorenal disorders were 0, 2, 2, 1, 0, 5, and 2 in the dominant (+) group and 2, 1, 2, 3, 3, 1, and 4 in the dominant (−) group. No statistically significant difference was found for the frequency distribution of the underlying disorders in the two groups showing different EEG patterns (chi-square = 9.285; *p* = 0.1582).

The mean frequency of the dominant rhythm was 7.2 ± 0.9 Hz in the 12 dominant (+) patients (minimum = 6 Hz; maximum = 9 Hz). The amplitude power of each wave band in the EEG, both with the eyes open and closed, is presented in Table 1. For the alpha band, two-way repeated ANOVA revealed that the dominant rhythm factor (+ vs. −) was significant (F (1, 26) = 5.481; *p* = 0.027). The power of the alpha rhythm in the dominant (+) participants was greater than that in the dominant (−) participants during both the eyes-open and eyes-closed conditions. For the theta rhythm, two-way ANOVA found that the interaction between the dominant rhythm factor and the eyes-open/closed factor was significant (F (1, 26) = 8.026; *p* = 0.009). The post hoc Sidak test revealed that the theta power during the eyes-closed condition in the dominant (+) participants was greater than in the dominant (−) participants (*p* = 0.009). No significant differences in the delta and beta bands were found using two-way ANOVA (*p* > 0.05). The differences between the power during the eyes-open and eyes-closed conditions were not significant for all bands in both the dominant (+) participants and the dominant (−) participants (*p* < 0.05).

### 3.2. Total ICDSC Score in Dominant (+) and Dominant (−) Participants

**Figure 2:**
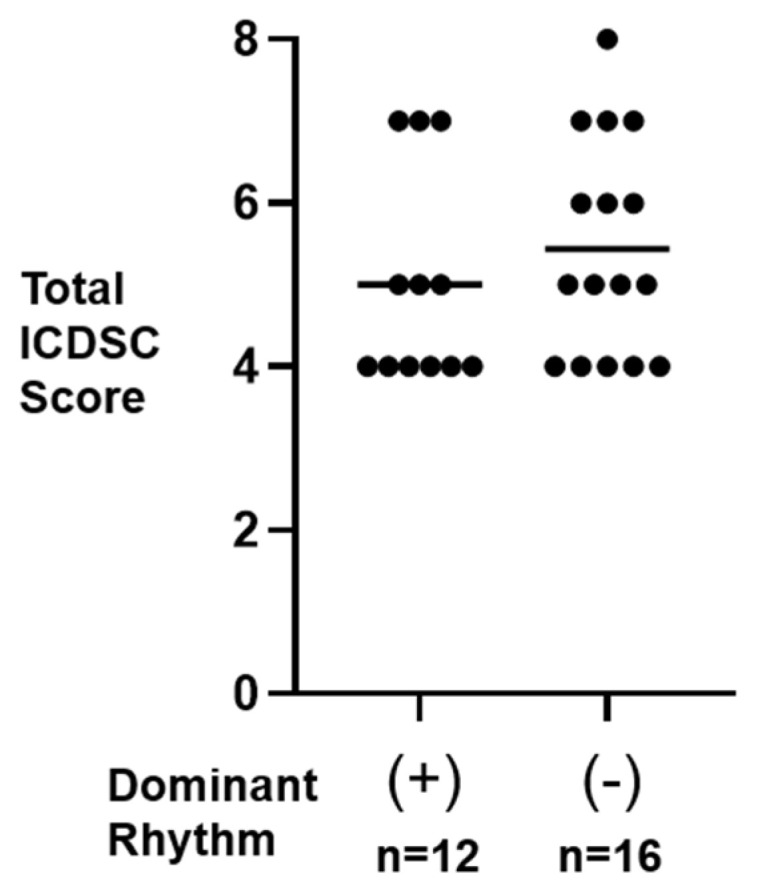
Total ICDSC scores of participants with (+) and without (−) dominant rhythm. Each filled circle indicates the individual data. The horizontal bar shows the average.

The distribution of the total ICDSC scores in both the dominant (+) and (−) participants is presented in Figure 2. The averaged total score (mean ± s.d.) was 5.0 ± 1.3 in the dominant (+) participants and was not statistically different from that in the dominant (−) participants (5.4 ± 1.3; Mann–Whitney U = 76.5; *p* = 0.36).

### 3.3. Individual ICDSC Scores and EEG Patterns

**Figure 3:**
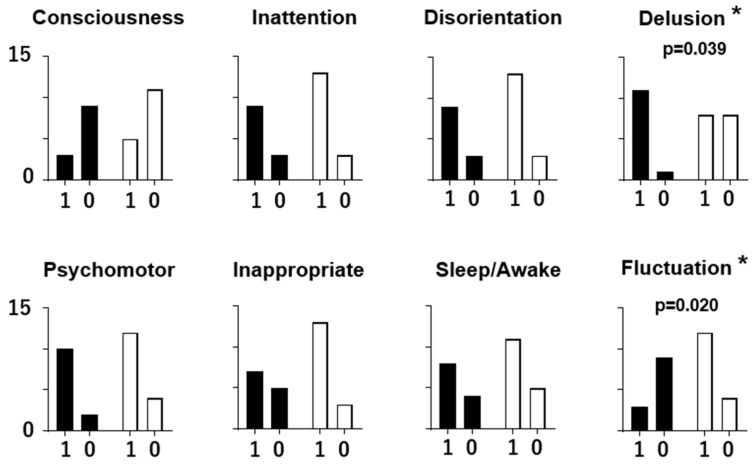
The numbers of participants scoring 1 or 0 for each ICDSC index: altered level of consciousness (Consciousness), inattention (Inattention), disorientation (Disorientation), hallucination or delusion (Delusion), psychomotor agitation or retardation (Psychomotor), inappropriate mood or speech (Inappropriate), sleep/wake cycle disturbance (Sleep/Awake), and symptom fluctuation (Fluctuation). The ICDSC score distribution (1 or 0) is shown as the number of participants with (black column) and without (white column) a dominant EEG rhythm. The differences were assessed using Fisher’s exact test. A significant difference (*p* < 0.05) is indicated as an asterisk at the right shoulder of the indices.

The relationship between the individual ICDSC scores and the EEG patterns is presented in Figure 3. It is indicated that ‘hallucination or delusion’ was more frequent in the dominant (+) participants than in the dominant (−) participants (Fisher’s exact test; *p* = 0.039), and ‘symptom fluctuation’ was more frequent in the dominant (−) participants than in the dominant (+) participants (*p* = 0.020). The other ICDSC scores show no statistically different distribution between the two groups (*p* > 0.05).

## 4. Discussion

### 4.1. EEG Characteristics in Delirious Participants

Two resting-state EEG patterns were observed in delirious patients in this study: the presence or absence of a dominant rhythm. The mean frequency in the dominant (+) participants was 7.2 Hz (ranging from 6 to 9 Hz). The power of the alpha band in the eyes-open and eyes-closed conditions and the power of the theta band in the eyes-closed condition in the dominant (+) participants were greater than those in the dominant (−) participants, indicating that the theta and alpha bands are dominant in dominant (+) delirious patients.

Furthermore, the diminution in the amplitude with eye opening, which is normally present, was not observed in the dominant (+) and dominant (−) delirious participants. The loss of the effects via eye opening/closing could be a common feature of delirium, as presented in the previous literature [12]. The absence of EEG reactivity to eye opening/ closing in delirium has been reported but has not been effectively utilized in distinguishing delirium [12,14,17,23]. The absence of a dominant rhythm has also been revealed as the characteristic EEG profile of delirium [12]. However, previous studies did not fully support the usefulness of these EEG findings in evaluating the pathophysiology of delirium [28,29]. This study clarifies that these two EEG features are related to distinct delirium symptoms and should be treated as different pathophysiological aspects of delirium.

### 4.2. Two Types of Delirium with Distinct Symptoms and EEG Patterns

This study found two resting-state EEG patterns: one showing a dominant rhythm at a slow alpha range without the effects of eye opening/closing and the other without a particular dominant rhythm. The total ICDSC score, usually used to evaluate the severity of delirium, showed no difference between the participants with the two EEG patterns (Figure 2). The severity of the delirious state is not mainly involved in the difference observed in EEG patterns.

Two of the eight delirium symptoms were found to be related to EEG patterns by analyzing the relation of each symptom in the ICDSC with the EEG pattern. ‘Hallucination or delusion’ was frequently observed in the patients whose EEG showed a dominant rhythm. Meanwhile, ‘symptom fluctuation’ often accompanied an EEG pattern without a dominant rhythm. The frequency of the other symptoms did not differ between the two groups.

Thus, the present study revealed two types of delirium: delirium accompanying a dominant EEG rhythm in the slow alpha range without attenuation during eyes opening, exhibiting hallucination or delusion, and delirium showing no dominant EEG rhythm with symptom fluctuation. The former and the latter types may correspond to hyperactive and hypoactive delirium, respectively. The present findings are consistent with the previous research indicating that hallucination and delusion are more frequently observed in the hyperactive delirium [8]. Hyperactive and hypoactive delirium could be diagnosed more clearly with EEG recordings. The treatment choices, including anti-psychotic use and adjusting day/night rhythm, may be adequately determined using these EEG classifications. This possibility should be assessed in future studies incorporating treatment outcomes.

There is also a possibility that the cognitive impairment in dominant (−) participants is more severe than that in the dominant (+) participants. The arousal level would be lower in the dominant (−) participants showing no basic EEG rhythm, which could be a sign of hypoactive delirium leading to cognitive impairment [9]. The presence of hallucination and delusion in the dominant (+) participants may indicate that the arousal level is maintained. The EEG pattern could switch from one to the other depending on the arousal level due to physical conditions.

### 4.3. Limitations

In this study, EEGs were measured once a day for ICDSC evaluation for a short duration. It is important to note that the two EEG patterns may not be directly linked to the symptomatic characteristics revealed by ICDSC, because ICDSC was scored on a 24-h basis in contrast to EEG being measured at a point of the day. Longer and repeated measurements should be informative and accurate in clarifying the pathophysiology of delirium; however, the clinical conditions of the delirious patients in this study made this difficult to realize.

This study’s limitations also include its small sample size. Analyses based on the underlying somatic disorders were not possible due to the small sample size in the present study. Especially, the underlying neurological disorders in the present study included cerebral infarction, hemorrhage, and Parkinson’s disease, which could directly affect brain functions. Future studies with a larger number of delirious patients would be necessary to validate the present findings. The analyses on the effects of sociodemographic data were not conducted in the present study and would also be important in future research. Heterogeneity of delirium would be further clarified using the abovementioned data in the future studies. The technical improvement of EEG measurements is also important. Steady EEG data are required to adequately utilize EEG signals to delineate EEG characteristics. Reducing movement artifacts will consolidate the present findings. The EEG amplitudes of delta and beta bands in the dominant (−) delirious participants were not different from those in the dominant (+) participants, supporting that the biological signals were correctly detected in the dominant (−) delirious participants.

Regarding behavioral interventions, this study employed eyes opening and closing. It was difficult to select tasks applicable to delirious patients with limited cognitive ability. However, it would be interesting to incorporate simple tasks other than eyes opening and closing to investigate different brain functions. Sensory-evoked potential recordings may also be interesting to investigate the cognitive function as well as to check the validity of EEG recording in the future studies.

Functional connectivity is also important to analyze in future research. Previous studies pointed out that loss of functional connectivity is a feature involved in the generation of delirium [30,31,32,33]. It would be interesting to record EEGs at multiple sites in delirious patients to analyze the relationship between EEG profiles and delirium symptoms. Higher-frequency bands, including gamma rhythm, are another area of interest [34].

Differentiation from dementia and depression is further required in clinical practice in the case of somatic diseases. Dementia and depression are commonly observed in patients admitted to a hospital for treatment of somatic disorders and should be differentiated from delirium to start adequate treatment [35,36]. Future studies are necessary to use the present findings to assess this issue.

## Figures and Tables

**Table 1:** The amplitude power values (μV^2^) of the delta, theta, alpha, and beta EEG ranges in the participants with (Dominant (+); n = 12) and without (Dominant (−); n = 16) a dominant rhythm (mean ± s.d.).

	Eyes	Delta	Theta	Alpha	Beta
Dominant (+)	Open	200.5 ± 399.6	50.0 ± 48.2	34.7 ± 18.9 ^a^	50.8 ± 52.1
	Closed	92.3 ± 68.5	64.3 ± 63.4 ^b^	36.7 ± 27.0 ^b^	39.1 ± 45.6
Dominant (−)	Open	134.9 ± 134.6	29.9 ± 34.2	18.1 ± 20.0	20.4 ± 24.4
	Closed	103.5 ± 92.8	27.0 ± 33.8	16.6 ± 17.9	17.1 ± 20.0

The two-way repeated-measures ANOVA with post hoc Sidak test indicated that the amplitude power in dominant (+) participants was significantly greater than that in the dominant (−) participants during the eyes-open (a) and eyes-closed (b) conditions (*p* < 0.05). No difference between the eyes-open and eyes-closed conditions was observed for the power of all EEG bands (*p* > 0.05).

## Data Availability

The data supporting the findings of this study are available from the corresponding author upon request. The data are not publicly available due to privacy and ethical restrictions.
